# Characterization and Analysis of 2-(2-Phenylethyl)-chromone Derivatives from Agarwood (*Aquilaria crassna*) by Artificial Holing for Different Times

**DOI:** 10.3390/molecules21070911

**Published:** 2016-07-13

**Authors:** Jin Ling Yang, Wen Hua Dong, Fan Dong Kong, Ge Liao, Jun Wang, Wei Li, Wen Li Mei, Hao Fu Dai

**Affiliations:** Hainan Key Laboratory for Research and Development of Natural Products from Li Folk Medicine, Key Laboratory of Tropical Crop Biotechnology, Ministry of Agriculture, Institute of Tropical Bioscience and Biotechnology, Chinese Academy of Tropical Agricultural Sciences, Haikou 571101, Hainan, China; jinlyang@126.com (J.L.Y.); dongwenhua@itbb.org.cn (W.H.D.); kongfandong@itbb.org.cn (F.D.K.); liaoge828@aliyun.com (G.L.); wangjun@itbb.org.cn (J.W.); liwei@itbb.org.cn (W.L.)

**Keywords:** artificial holing agarwood, *Aquilaria crassna*, HPLC/DAD/ESI/MS/MS, 2-(2-phenylethyl) chromone derivatives

## Abstract

A total of fifty-six chromones, including seven 5,6,7,8-tetrahydro-2-(2-phenylethyl)-chromones (THPECs), five 5,6-epoxy-2-(2-phenylethyl)chromones (EPECs), seven 5,6:7,8-diepoxy-2-(2-phenylethyl)chromones (DEPECs) and thirty-seven 2-(2-phenylethyl)chromones of the flidersia type (FTPECs), were characterized by HPLC/DAD/ESI/MS/MS in three agarwood samples (from *Aquilaria crassna*) induced by artificial holing with different holing times. The characteristic fragmentation behavior of DEPECs and EPECs, and the methods to distinguish these four types of chromones by MS analysis were described for the first time. In addition, it was found that the relative contents of DEPECs and EPECs were down-regulated, while the relative contents of THPECs and FTPECs were up-regulated for the samples from two, four and five years of the agarwood formation time. However, the relative contents of six most widespread and abundant FTPECs presented roughly upward based on the formation time. These results could be referenced to distinguish different agarwood samples collected from different formation time.

## 1. Introduction

Agarwood is the dark resinous and aromatic wood from genus *Aquilaria* trees belonging to the family Thymelaeaceae [1,2,3], and is created as a tree response to various form of injury, including natural injuries, such as lightning strikes, animal grazing, insect attacks or microbial invasion, or artificial injuries, such as cutting, nailing, holing, fire, chemical wounding, and deliberate fungal inoculation [3,4,5]. Agarwood not only plays an important role in Traditional Chinese Medicine, but also has been used for centuries as incense in Buddhist, Hindu and Islamic ceremonies [1,2,3]. Unfortunately, in natural forests, only 7%–10% of the agarwood trees contain resinous material, and wild agarwood is rare due to its exhaustive exploitation. Nowadays, agarwood in trade mostly comes from cultivated *Aquilaria* trees induced by artificial methods, of which the traditional artificial holing is the most common and popular one. As far as we know, nineteen accepted species have been reported up to date, growing from southeast Asia to the Malay Archipelago [1,2]. However, only the heartwood of *A. crassna*, *A. malaccensis*, *A. sinensis* and *A. filarial* have been commercially exploited [1]. 2-(2-Phenylethyl)chromone derivatives, of which about one hundred different structures have been reported, are considered to be one of the characteristic and most abundant constituents responsible for the quality of agarwood [1,3,4,6,7,8,9]. These compounds were divided into four types according to the various chromone moiety skeletons, in which the benzyl moiety is the same. In these four types, both 2-(2-phenylethyl)chromones of the flidersia type (FTPECs) and 5,6,7,8-tetrahydro-2-(2-phenylethyl)chromones (THPECs) were widely distributed and accumulated most in agarwood, while only four 5,6-epoxy-2-(2-phenylethyl)chromones (EPECs) and three 5,6:7,8-diepoxy-2-(2-phenylethyl) chromones (DEPECs) with one or two epoxy substituents on the chromone moiety respectively, have been reported from 2005 to the present [7,10,11].

Based on the above reports, 2-(2-phenylethyl)chromones were considered as the necessary diagnostic components to evaluate the quality of agarwood. Mei et al. summarized the MS characterization of the FTPECs, which was helpful for the analysis and characterization of FTPECs in agarwood by GC-MS [3]. Xia et al. reported that 29 FTPECs were detected from agarwood by supercritical fluid chromatography in combination with mass spectrometry (SFC-MS) then obtained further detailed structural information by using tandem mass spectrometry (MS/MS) [12]. Li et al. identified and quantified eight characteristic THPECs in Chinese eaglewood, and analyzed the MS fragmentation behavior of THPECs by HPLC/DAD/MS [13]. Lancaster et al. detected diagnostic ions at *m/z* 319.118 (FTPECs) or 349.129 (FTPECs) and the occurrence of ten or more of the other target chromone ions by real time time-of-flight mass spectrometry (DART-TOFMS) to infer agarwood [5]. Espinoza et al. analyzed the diagnostic chromones ions by using DART-TOFMS followed by discriminant analysis to differentiate wild agarwood from cultivated samples [2]. However, the MS characterization of EPECs and DEPECs have not been reported yet. In this paper, the MS characterization and fragmentation behavior of EPECs and DEPECs was described, and the fragmentation regularity of mass spectra (MS) used to distinguish and identify the four different types of 2-(2-phenylethyl)chromones was concluded for the first time.

Nowadays, artificial holing agarwood from *A. crassna* tree plays an extremely important role in the market. In this report, we detected and analyzed 2-(2-phenylethyl)chromones in three agarwood samples (from *A. crassna*) with different holing times by using HPLC/DAD/ESI/MS^2^, and revealed the variation of relative contents of different types of 2-(2-phenylethyl)chromones in agarwood after two, four and five years of artificial holing. This could be referenced for the variation of 2-(2-phenylethyl)chromones, also identification and quality evaluation of agarwood with different formation time.

## 2. Results and Discussion

### 2.1. Characteristic Fragmentation Behavior of Reference Compounds ***F1**–**F31***

**Figure 1:**
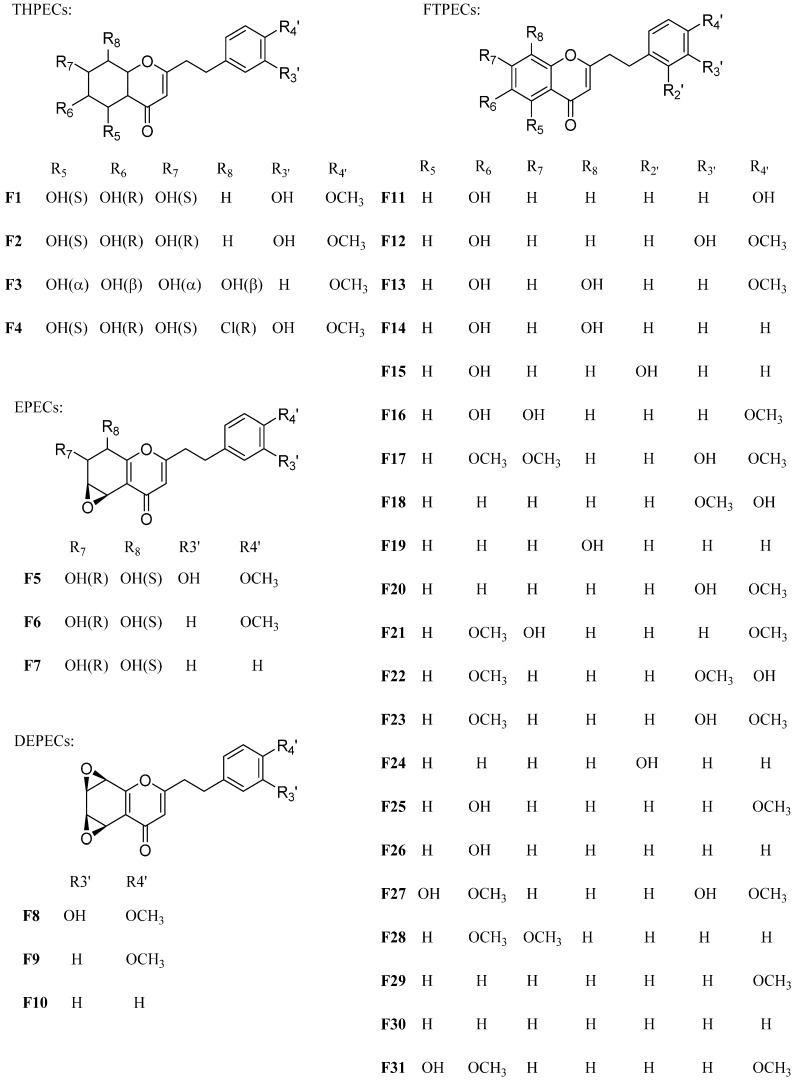
The structure of reference compounds **F1**~**F31**.

In this study, the different fragmentation behavior of four types of chromones according to their MS spectra was summarized by the reference compounds (Table 1 and Figure 1). The characteristic fragment ions produced by four types of chromones due to their structural difference on the basic skeleton of chromone moiety were used as the basis for identification. For THPECs (compounds **F1**–**F4**), the characteristic fragmentations were the loss of a molecule of H_2_O ([M + H–18]^+^) and a successive loss of another H_2_O molecule ([M + H–18–18]^+^) [13]. For EPECs (compounds **F5**–**F7**), the characteristic fragmentations were loss of a molecule of H_2_O ([M + H–18]^+^), followed by loss of a molecule of CO ([M + H–18–28]^+^). For DEPECs **F8**–**F10**, the characteristic fragmentation was the loss of a molecule of CO ([M + H–28]^+^).

**Figure 2:**
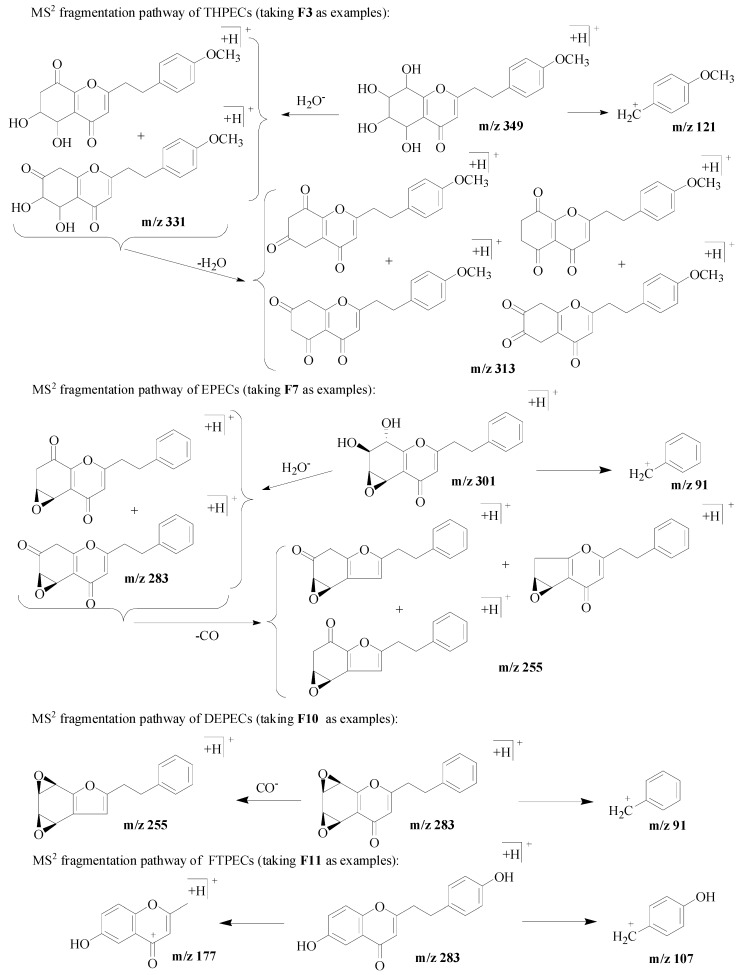
MS^2^ fragmentation pathway of four types of chromones.

For twenty-one FTPECs in the reference compounds, their characteristic fragmentations were benzyl ions and/or chromone moiety ions. In addition, the benzyl ions observed in the MS spectrum of these four types chromones were the same, and the typical ions included *m/z* 91 [C_7_H_7_]^+^, 107 [C_7_H_6_ + OH]^+^, 121 [C_7_H_6_ + OCH_3_]^+^, 137 [C_7_H_5_ + OH + OCH_3_]^+^, 151 [C_7_H_5_ + 2OCH_3_]^+^. Figure 2 presents examples of the MS^2^ fragmentation behavior from each type of chromone.

### 2.2. The Method Deduced for Identification of Four Types of 2-(2-Phenylethyl)chromone Derivatives

**Figure 3:**
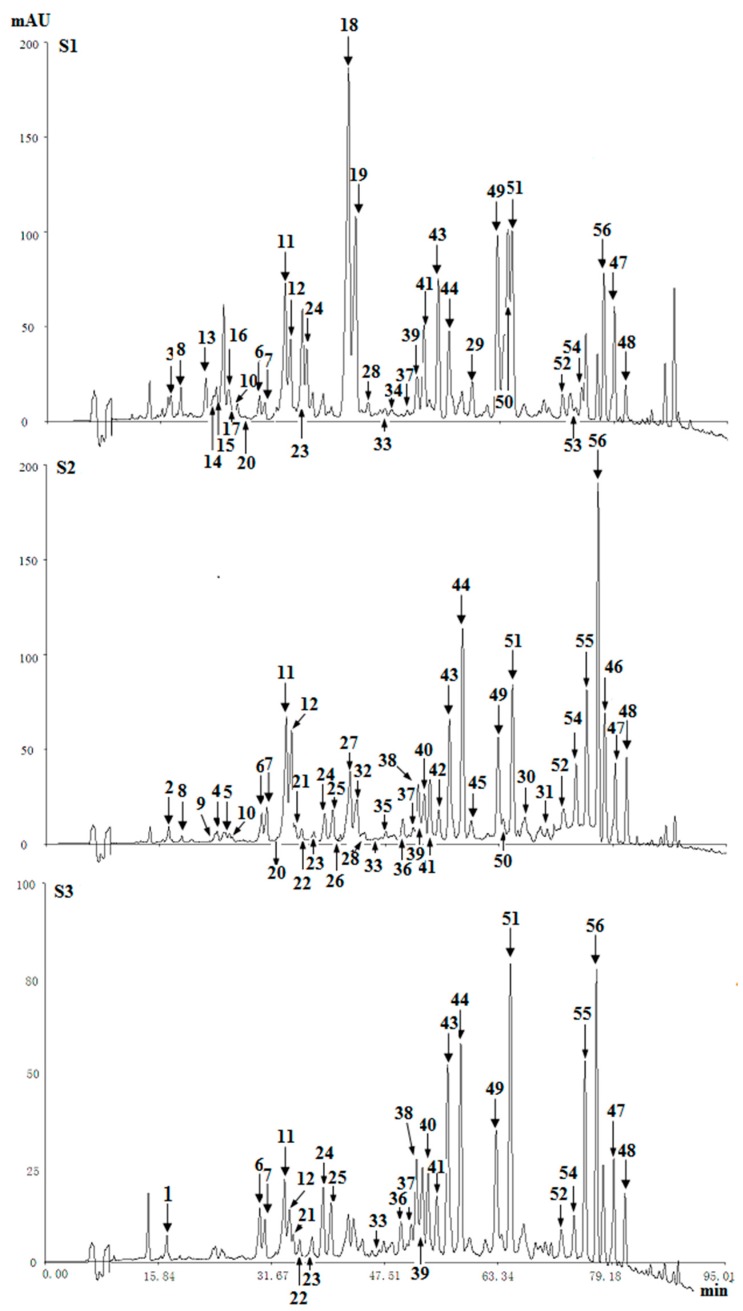
Chromatograms of three agarwood samples (S1, S2, S3) at 254 nm.

(1) The type of 2-(2-phenylethyl)chromone could be determined by the characteristic fragment ions as discussed above and listed in Figure 3. The present of the ions [M + H–18]^+^ and [M + H–18–18]^+^ was proposed to be THPECs. When both ions [M + H–18]^+^ and[M + H–28]^+^ were observed in the spectrum, EPECs was deduced. If only [M + H–28]^+^ was detected, it would be DEPECs. FTPECs only gave the peaks of benzyl ions and/or chromone moiety ions in the MS spectrum. It should be noticed that the [M + H–18]^+^ ion may appear as a pseudo-characteristic ion during identification, when the CH_2_-CH_2_ group between chromone moiety and phenyl moiety was substituted by a hydroxyl group (-OH) [1].
(2) The four types of 2-(2-phenylethyl)chromone derivatives in agarwood have different basic skeletons (the epoxy group was assigned as a part of the basic skeletons), but substituted with similar substituent groups (mainly hydroxyl, methoxyl group and/ or chlorine atom). The molecular weights of the basic skeletons of THPECs, EPECs, DEPECs and FTPECs were 254, 268, 282 and 250, respectively. Thus, the number of different substituents of the structure could be deduced by Formula (1), where “MW” meant the molecular weight, “a” meant the number of methoxy groups, “b” represented the number of hydroxyl groups, and “c” meant the number of chlorine atom.
  (1) $$\mathrm{MW}-(30a+16b+34c)=\left\{ \begin{array}{l} 254\;\left(THPECs\right)\\ 268\;\left(EPECs\right)\\ 282\;\left(DEPECs\right)\\ 250\;\left(FTPECs\right) \end{array}\right.$$
(3) The four types of chromones presented the same characteristic benzyl ion, while it could not be observed in some compounds. The benzyl moiety without substituent group was 91 [C_7_H_7_]^+^, therefore, the number of hydroxyl and/or methoxyl groups substituted on the benzyl moiety could be deduced according to Formula (2), where “MW_bm_” means the molecular weight of benzyl ion, “a_bm_” means the number of methoxy groups on the benzyl moiety, “b_bm_” represents the number of hydroxyl groups on the benzyl moiety. For example, according to Formula (2), the hydroxyl- or methoxyl-substituted benzyl moiety provided characteristic ions at *m/z* 107 [C_7_H_6_ + OH]^+^, 121 [C_7_H_6_ + OCH_3_]^+^, 137 [C_7_H_5_ + OH + OCH_3_]^+^, 151 [C_7_H_5_ + 2OCH_3_]^+^:
MW_bm_ − 91 = 30a_bm_ + 16b_bm_ (2)
(4) Except for FTPECs, the fragment ion of the chromone moiety was rarely observed in the MS^2^ spectra of THPECs, EPECs and DEPECs, nevertheless the number of substituent groups of the chromone moiety could still be calculated by subtracting the number of substituents on the benzyl moiety deduced by Formula (2) from the number of substituents on the whole structure deduced by Formula (1).
  For FTPECs with hydroxyl-substituted benzyl moieties, according to [3,6], if one of characteristic fragment ions at *m/z* 161 [C_10_H_8_O_2_ + H], 177 [C_10_H_7_O_2_ + OH + H], 191 [C_10_H_7_O_2_ + OCH_3_ + H], 193 [C_10_H_6_O_2_ + 2OH + H], 207 [C_10_H_6_O_2_ + OCH_3_ + OH + H], 221 [C_10_H_6_O_2_ + 2OCH_3_ + H] from different substituted chromone moieties was observed, a 4′/2′-OH substituent on the benzyl moiety was deduced. Otherwise, if none of them was observed, a hydroxyl group substituted at the 3′ position of the benzyl moiety was deduced, whether an ion of chromone moiety at *m/z* 160, 176, 190, 192, 206, 220 appeared or not. The above mentioned MS characterization has been confirmed by the FTPEC reference compounds in Table 1. The observed fragment ions of the chromone moieties of **F11**, **F15**, **F18**, **F22** and **F24** with 4′/2′-OH on the benzyl moiety were *m/z* 177, 177, 161, 191 and 161, respectively, which were not observed for the **F12**, **F17**, **F20**, **F23** and **F27** with 3′-OH on the benzyl moiety, while only a fragment ion at *m/z* 220 of **F17** was observed. These chromone moiety ions could be used to suggest the position of hydroxyl groups on the benzyl moieties.
(5) Following the above procedure, a compound could be characterized as one of the four types of 2-(2-phenylethyl)chromones, as well as calculated the numbers of substituent groups of the chromone moiety and benzyl moiety. Furthermore, the position of substituent groups could be deduced sometimes. Besides, if more than one structure were suggested for a single peak, the comparison of retention time and MS spectra with reference compounds was performed. The compound was identified when the data were the same to the reference compound, otherwise, it might be a new compound.

### 2.3. Identification of 2-(2-Phenylethyl)chromones According to MS Characterization

In the HPLC chromatograms of the ether extract of three agarwood samples (S1, S2, S3) detected at UV 254 nm, a total of fifty-six compounds (four types of chromones) were detected (Figure 3). By comparing their retention time and MS spectra with reference compounds, twenty-six of them were identified, and thirty compounds were tentative identified. The identified or characterized results of THPECs, EPECs and DEPECs were listed in Table 2, and the retention time of THPECs, EPECs and DEPECs were in the range of 12~30 min, 18~34 min, 21~43 min, respectively. The results of FTPECs were showed in Table 3, and the retention time of FTPECs were in the range of 34~80 min, except for compound **20** (26.1 min).

#### 2.3.1. Structural Analysis of THPECs

Taking compounds **1** and **3** for example, the characteristic fragment ions at *m/z* 331([M + H–18]^+^) and 313 ([M + H–18–18]^+^), indicated both were THPECs. The molecular weight (MW) of these two compounds were determined as 348 based on the protonated precursor ion at *m/z* 349. According to formula (1) (MW − (30a + 16b +34c) = 254), a, b and c were calculated as 1, 4 and 0, respectively, which meant compounds **1** and **3** had four hydroxyl and one methoxyl substituent groups on the whole compound. Furthermore, the benzyl ion at *m/z* 121 was found in compound **1**, which meant one methoxy substitution occurred on its benzyl moiety according to Formula (2), thus, the other four hydroxyl substitutions occurred on its chromone moiety. Neither benzyl ion nor chromone moiety ions were observed in compound **3**, thus the positions of substituent groups were uncertain. By searching the literature, we found six THPECs with the MW = 348 were reported, including four of them possessing one methoxy substitution occurring on the benzyl moiety, and four hydroxyl substitutions occurring on their chromone moiety, which matched the above deduced structural characterization of compound **1**, so compound **1** was tentative identified as one of the four reported compounds. The positions of the substituent groups of compound **3** were uncertain, so it was tentative identified as one of the six reported compounds while different from compound **1**. By comparing the retention time and MS spectra with the reference compounds, compound **1** was identified as **F3**, and further proved that the above identification method was reasonable.

According to above method of identification, compounds **1**–**7** were deduced as THPECs. Except for compound **5** (*m/z* 317), the other six compounds, with [M + H]^+^ ions of *m/z* 319, 303, 367, 337, have been reported as THPECs. Compound **5** was identified as a new compound.

#### 2.3.2. Structural Analysis of EPECs

Taking compounds **10** and **11** for example, according to the characteristic fragment ions of *m/z* 313 ([M + H–18]^+^) and 285 ([M + H–18–28]^+^), they were identified as EPECs. The protonated precursor ions at *m/z* 331 meant their molecular weight (MW) was 330. According to the Formula (1), a, b and c were calculated as 1, 2 and 0, respectively, which meant they had two hydroxyl and one methoxyl substitutions on the whole compound. The benzyl ion at *m/z* 121 indicated one methoxy substitution occurred on its benzyl moiety according to Formula (2), therefore, the two hydroxyl groups substitutions occurred on the chromone moiety. Subsequently, by literature searching, only one EPEC with MW = 330 was reported, which meant that one of them would be a new compound. By comparing the retention time and MS spectra with reference compound, compound **11** was identified as **F6**, which proved that the above identification method was feasible, and meanwhile compound **10** should be a new compound.

By the same method, compounds **8**–**12** were deduced as EPECs. Except for compound **9** (which [M + H]^+^ ion is *m/z* 285), all the other compounds, which [M + H]^+^ ions were at *m/z* 347 or 301, have been reported. By comparing retention time and MS spectra with reference compounds, compounds **8** and **12** were identified as reference compounds **F5** and **F7**, respectively. Compounds **9** and **10** were proposed to be new compounds as listed in Table 2, because only four EPECs have been reported until now [7,14].

#### 2.3.3. Structural Analysis of DEPECs

According to the above method of identification, compounds **13**–**19** were assigned as DEPECs. By comparing the retention time and MS spectra with the reference compounds, compounds **16**, **18**, and **19** were identified as the reference compounds **F8**~**F10**, respectively. The other DEPECs were proposed to be new compounds, as only a total of three DEPECs (**F8**~**F10**) have been reported until now [10]. The results are shown in Table 2.

#### 2.3.4. Structural Analysis of FTPECs

Taking compounds **22**~**24**, **27** and **30** for example, only benzyl ions (*m/z* 137 and 121) and chromone moiety ion (*m/z* 177) were detected, leading to identification of these compounds as FTPECs. All of them showed protonated precursor ions at *m/z* 313, which meant their molecular weight (MW) was 312. According to Formula (1), the a, b and c values were calculated as 1, 2 and 0, respectively, which meant they have two hydroxyl and one methoxyl substituent group on the whole compound. Then, according to Formula (2), the benzyl ion at *m/z* 137 was found in compounds **22** and **23**, which meant one methoxyl and one hydroxyl substitution occurred on their benzyl moiety, while the benzyl ion at *m/z* 121 was detected in compounds **24**, **27** and **30**, which meant one methoxy substitution occurred on their benzyl moiety. Consequently, one hydroxyl substitution occurred on the chromone moiety of compounds **22** and **23**, and two hydroxyl substitutions occurred on the chromone moiety of compounds **24**, **27** and **30**. In particular a chromone moiety ion (*m/z* 177) was detected in compound **22**, which indicated the hydroxyl group should be substituted at the 4′/2′ position of the benzyl moiety. Five FTPECs with MW = 312 have been traced from the literature, so the five FTPECs were tentatively identified as reported compounds. By comparing the retention time and MS spectra with reference compounds, compounds **22**, **24** and **27** were identified as **F12**, **F13** and **F16**, respectively, and the results proved that the above identification method was reasonable.

According to the method of identification, compounds **20**–**56** were deduced as FTPECs (Table 3), among which nineteen compounds were identified according to the reference compounds.

Finally, fifty-six 2-(2-phenylethyl)chromone derivatives were identified or characterized from the three samples according to the fragmentation behavior and the comparison of retention time and MS spectra with reference compounds (Table 1). A total of 37 (seven DEPECs, four EPECs, three THPECs and 23 FTPECs), 43 (five EPECs, five THPECs and 33 FTPECs), and 29 (one DEPEC, two EPECs, three THPECs and 23 FTPECs) 2-(2-phenylethyl)chromones were identified or characterized in S1, S2 and S3, respectively. The respective relative content of 2-(2-phenylethyl)chromone derivatives was 66.42%, 81.39% and 79.20% in S1, S2 and S3 (Table 4).

**Figure 4:**
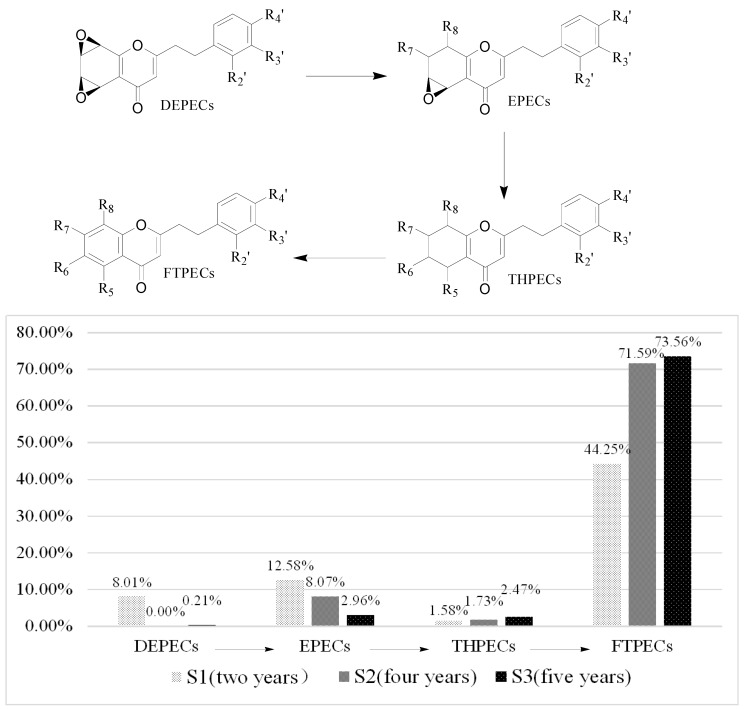
Biosynthetic progress of chromones and the varieties of the four types of chromones in S1 to S3.

Based on the above data, it was found that the number and relative content of DEPECs (7/8.01%) from S1 was the highest among the three samples, while they were merely traces in S2 and S3 (Table 4, Figure 4). The relative content of EPECs showed a downtrend from S1 to S3 (12.58%, 8.07% and 2.96%, respectively), while the relative content of THPECs and FTPECs showed an uptrend from S1 to S3 (1.58%/1.73%/2.47%, 44.25%/71.59%/73.56%, respectively). It was noticeable that both the total number and relative content of DEPECs, and EPECs decreased obviously (20.59%, 8.07% and 3.17%, respectively) from S1 to S3, and this was consistent with that observed in the HPLC chromatograms (Figure 3), in which the height of peaks for the DEPECs, EPECs (before 43 min) showed a remarkable decreasing trend. This finding was in agreement with the proposed biosynthetic pathways of the four types of chromones (Figure 4) [11], which showed transformations among them during different agarwood formation time. DEPECs have been recognized as precursors, and only accumulated at the early stage of agarwood formation. Then, the next group was EPECs, where the low occurrence rate also suggested they were early intermediates during the agarwood formation. The following group was the highly oxidized THPECs, and the last presented group were FTPECs, both of which were widely distributed in agarwood.

In total, twenty-one 2-(2-phenylethyl)chromones (two EPECs, two THPECs and 17 FTPECs) were detected in all three samples, while ten (six DEPECs, one THPEC and three FTPECs), twelve (one EPEC, three THPECs and eight FTPECs), and one (THPEC) 2-(2-phenylethyl)chromone were only found in S1, S2, and S3, respectively. Furthermore, we found that the relative content of six FTPECs, 6,8-dihydroxy-2-[2-(4-methoxy)phenylethyl]chromone, 6-methoxy-7-hydroxy-2-[2-(4-methoxy)-phenylethyl]chromone, 6-hydroxy-2-(2-phenylethyl)chromone, 6,7-dimethoxy-2-(2-phenylethyl) chromone, 2-[2-(4-methoxy)phenylethyl]chromone, and 2-(2-phenylethyl)chromone, which are reported as the main constituents of agarwood, showed a uptrend from S1 to S3. This finding suggested that the relative content of the six 2-(2-phenylethyl)chromones was the significant factor to evaluate the formation time of agarwood.

## 3. Experimental Section

### 3.1. Chemicals and Materials

**Figure 5:**
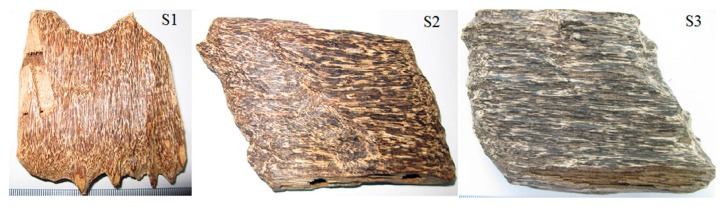
Pictures of the three batches of agarwood.

HPLC-grade acetonitrile (ACN) and methanol were supplied by Tedia (Fairfield, CR, USA). Chromatographic grade absolute formic acid was purchased from Roe Scientific Inc. (Shanxi, HPLC, China). Water was purified using a Milli-Q Plus185 system from Millipore (Milford, MA, USA). The times of artificial holing into the trunk of *A. crassna* tree were Aug of 2011 (S1), 2009 (S2) and 2008 (S3), respectively, so the agarwood formation times of S1–S3 were 2 years, 4 years and 5 years, respectively (Figure 5). The samples were collected by Dr. Haofu Dai from Guangnan Province of Vietnam at 14 August 2013. Voucher specimens were deposited at Institute of Tropical Bioscience and Biotechnology, Chinese Academy of Tropical Agricultural Sciences.

Thirty-one references compounds were isolated and purified from agarwood in our previous work [11,14,15,16,17]. Their structures were identified by spectroscopic methods (MS and NMR), and they are listed in Table 1 and Figure 1. The purity of each compound was determined to be higher than 97% by high-performance liquid chromatography (HPLC).

### 3.2. Preparation of Sample Solutions and Reference Compound Solutions

In this study, the crushed agarwood (100g, dry weight) was soaked in Et_2_O (3 × 200 mL) and extracted by ultrasonication (3 × 15 min). The Et_2_O extracts were filtered and evaporated to get brownish yellow oil. The yields of S1, S2 and S3 were 0.56%, 0.46%, and 2.08%, respectively. Then each extract oil sample was diluted to 1 mg/mL with methanol in a volumetric flask. The solution was filtered through 0.45 µm membranes and stored at 4 °C until use. Accurately weighed samples of thirty-one reference compounds were dissolved in methanol (1.0 mg/mL), respectively, and filtered through 0.45 µm membranes to get the standard solutions. All standard solutions were stored at 4 °C until use.

### 3.3. HPLC Chromatographic Condition

A Dionex 3000 series HPLC instrument composed of a diode array detector, a vacuum degasser, a quaternary pump and an auto-sampler was used (Bruker Daltonics Inc., Bremen, HPLC/DAD/VD/QP/AS, Germany). The chromatographic separation was carried out on a Dionex-Acclaim 120 C_18_column (250 mm × 4.6 mm, 5 µm). The mobile phase consisted of acetonitrile (A) and water-acetic acid (99.5:0.5, *v*/*v*) (B). The gradient elution program was as follows: 25%–55% A in 0–60 min, 55%–80% A in 60–80 min, 80%–100% A in 80–90 min, and 100% A in 90–95 min. The flow rate was 0.4 mL/min, the column temperature was maintained at 26 °C, and the injection volume was 20 µL. The detection wavelength was set at 254 nm for all the tested compounds. 

### 3.4. MS Spectrometry

MS experiments were performed using an electrospray ionization tandem mass spectrometry system (Amazon SL, Bruker Daltonics Inc., Bremen, Germany) mainly in positive-ion mode. Helium gas was used as the collision gas and high-purity nitrogen gas as the nebulizer and drying gas at flow rates of 0.4 L/min and 6.0 L/min, respectively. The ESI source conditions were as follows: capillary voltage, −4000 V (position); end plate voltage, −500 V (position); drying gas temperature of 250 °C; and nebulizer pressure 15 psi. Scan spectra from *m/z* 70 to 2200. All the mass data was processed using Bruker Compass Data Analysis 4.0 software (Bruker Daltonics: Germany, 2009).

## 4. Conclusions

HPLC/ESI-MS/MS is a powerful tool to identify or characterize 2-(2-phenylethyl)chromone derivatives in agarwood. Thirty-one reference compounds including four types of chromones were analyzed by ESI-MS/MS, thus the characteristic fragmentation behavior of DEPECs and EPECs and the methods to distinguish the four types of chromones by their MS characteristic fragmentations were described for the first time, on the basis of the MS spectra of the thirty-one reference compounds. A total of fifty-six 2-(2-phenylethyl)chromone derivatives comprising seven DEPECs, five EPECs, seven THPECs and thirty-seven FTPECs were identified or characterized by their fragmentation pathways and characteristic fragment ions. It was found, with the increase of artificial holing time of agarwood (two years to five years), the total relative content of DEPECs and EPECs showed a downtrend, while the relative content of THPECs and FTPECs showed an increasing trend, which was consistent with the biosynthetic pathway of the formation of agarwood. The relative content of six FTPECs went upward from S1 to S3. This finding could be useful to distinguish agarwood samples with different formation times, and further studies on more agarwood samples with different formation times are needed to validate these conjectures.

## Figures and Tables

**Table 1:** Characterization of thirty-one reference compounds by HPLC/ESI-MS/MS.

Type	No.	t_R_/min	Fragment Ions (*m/z*)						Name
			[M + H]^+^	[M + H–18]^+^	[M + H–18–18]^+^	[M + H–18–28]^+^	CM	BM	
THPECs	**F1**	12.9	349	331	313			137	(5S*,6R*,7S*)-5,6,7-Trihydroxy-2-[2-(3-hydroxy-4-methoxyphenyl)ethyl]-5,6,7,8-tetrahydrochromone
	**F2**	13.2	349	331	313			137	(5S*,6R*,7R*)-5,6,7-Trihydroxy-2-[2-(3-hydroxy-4-methoxyphenyl)ethyl]-5,6,7,8-tetrahydrochromone
	**F3**	15.2	349	331	313			121	*5α,6β,7α,8β-Tetrahydroxy-2-[2-(4-methoxyphenyl)-ethyl]-5,6,7,8-tetrahydrochromone*
	**F4**	17.3	383	365	347			137	(5S*,6R*,7S*,8R*)-8-Chloro-5,6,7-trihydroxy-2-[2-(3-hydroxy-4-methoxyphenyl)ethyl]-5,6,7,8-tetrahydro-chromone
EPECs	**F5**	18.3	347	329		301		137	*5,6-Edroxy-7,8-dihydroxy-2-[2-(3-hydroxy-4-methoxy)phenylethyl]-5,6,7,8-tetrahydrochromone*
	**F6**	33.1	331	313		285		121	*5,6-Edroxy-7,8-dihydroxy-2-[2-(4-methoxy)phenyl-ethyl]-5,6,7,8-tetrahydrochromone*
	**F7**	33.8	301	283		255		91	*5,6-Edroxy-7,8-dihydroxy-2-(2-phenylethyl)-5,6,7,8-tetrahydrochromone*
DEPECs	**F8**	24.4	329			301		137	*5,6:7,8-Diedroxy-2-[2-(3-hydroxy-4-methoxy)phenyl-ethyl]-5,6,7,8-tetrahydrochromone*
	**F9**	42.6	313			285		121	*Oxidoagarochromones B*
	**F10**	43.5	283			255		91	*Oxidoagarochromones A*
FTPECs	**F11**	34.7	283				177	107	*6-Hydroxy-2-[2-(4-hydroxy)phenylethyl]chromone*
	**F12**	36.6	313					137	*6-Hydroxy-2-[2-(3-hydroxy-4-methoxy)phenylethyl]-chromone*
	**F13**	38.3	313				192	121	*6,8-Dihydroxy-2-[2-(4-methoxy)phenylethyl]chromone*
	**F14**	39.5	283				192	91	*6,8-Dihydroxy-2-(2-phenylethyl)chromone*
	**F15**	41.5	283				177	107	*6-Hydroxy-2-[2-(2-hydroxy)phenylethyl]chromone*
	**F16**	42.4	313					121	*6,7-Dihydroxy-2-[2-(4-methoxy)phenylethyl]chromone*
	**F17**	43.2	357				220	137	*6,7-Dimethoxy-2-[2-(3-hydroxy-4-methoxy)phenyl-ethyl]chromone*
	**F18**	47.5	297				161	137	*2-[2-(3-Methoxy-4-hydroxy)phenylethyl]chromone*
	**F19**	49.8	267				176	91	*8-Hydroxy-2-(2-phenylethyl)chromone*
	**F20**	50.4	296					137	*2-[2-(3-Hydroxy-4-methoxy)phenylethyl]chromone*
	**F21**	51.2	327					121	*6-Methoxy-7-hydroxy-2-[2-(4-methoxy)phenylethyl]-chromone*
	**F22**	52.3	327				191	137	*6-Methoxy-2-[2-(3-methoxy-4-hydroxy)phenylethyl]-chromone*
	**F23**	54.3	327					137	*6-Methoxy-2-[2-(3-hydroxy-4-methoxy)phenylethyl]-chromone*
	**F24**	55.2	267				161	107	*2-[2-(2-Hydroxy)phenylethyl]chromone*
	**F25**	56.5	297					121	*6-Hydroxy-2-[2-(4-methoxy)phenylethyl]chromone*
	**F26**	58.3	267				176	91	*6-Hydroxy-2-(2-phenylethyl)chromone*
	**F27**	59.1	343					137	*5-Hydroxy-6-methoxy-2-[2-(3-hydroxy-4-methoxy)-phenylethyl]chromone*
	**F28**	64.9	311				220	91	*6,7-Dimethoxy-2-(2-phenylethyl)chromone*
	**F29**	72.0	281					121	*2-[2-(4-Methoxy)phenylethyl]chromone*
	**F30**	73.8	251				160	91	*2-(2-Phenylethyl)chromone*
	**F31**	78.7	326					121	*5-Hydroxy-6-methoxy-2-[2-(4-methoxy)phenylethyl]-chromone*

CM: chromone moiety; BM: benzyl moiety.

**Table 2:** The MS characterization and identified or characterized results of DEPECs, EPECs and THPECs from S1 to S3.

No.	t_R_ (min)	[M + H]^+^ (*m/z*)	(+)Fragment Ions (*m/z*)					OCH_3_	OH	Identification	RFC	RC (%)		
			[M + H–18]^+^	[M + H–18–18]^+^	[M + H–18–28]^+^	CM	BM					S1	S2	S3
THPECs														
**1**	15.9	349	331	313			121	1	4	*5α,6β,7α,8β-Tetrahydroxy-2-[2-(4-methoxyphenyl)ethyl]-5,6,7,8-tetrahydrochromone*	**F3**			0.51
**2**	16.0	319	301	283					4	*(5S,6S,7R)-5,6,7-Trihydroxy-2-[2-(2-hydroxyphenyl)-ethyl]-5,6,7,8-tetrahydrochromone or isomers*			0.15	
**3**	16.7	349	331	313				1	4	*Isomer of **F3***		0.50		
**4**	23.7	303	285	267					3	*rel-(5R,6S,7R)-5,6,7-Trihydroxy-2-(2-phenylethyl)-5,6,7,8-tetrahydrochromone*			0.09	
**5**	24.2	317	299	281			121	1	2	*New compound (two hydroxy on CM, one methoxy on BM)*			0.08	
**6**	29.4	367	349	331			121	1	3	*rel-(5R,6S,7S,8R)-8-Chloro-5,6,7-trihydroxy-2-[2-(4-methoxyphenyl)ethyl]-5,6,7,8-tetrahydrochromone*		0.65	0.65	1.16
**7**	30.1	337	319	301					3	*(5S,6S,7S,8R)-8-Chloro-2-(2-phenylethyl)-5,6,7-trihydroxy-5,6,7,8-tetrahydrochromone or new compound*		0.43	0.76	0.80
EPECs														
**8**	18.5	347	329		301		137	1	3	*5,6-Edroxy-7,8-dihydroxy-2-[2-(3-hydroxy-4-methoxy)-phenylethyl]-5,6,7,8-tetrahydrochromone*	**F5**	1.13	0.12	
**9**	23.3	285	267		239				1	*New compound (one hydroxy on CM)*			0.09	
**10**	25.0	331	313		285		121	1	2	*New compound (isomer of F6)*		0.94	0.15	
**11**	33.0	331	313		285		121	1	2	*5,6-Edroxy-7,8-dihydroxy-2-[2-(4-methoxy)phenylethyl]-5,6,7,8-tetrahydrochromone*	**F6**	7.25	4.95	2.11
**12**	33.7	301	283		255				2	*5,6-Edroxy-7,8-dihydroxy-2-(2-phenylethyl)-5,6,7,8-tetrahydrochromone*	**F7**	3.26	2.76	0.85
DEPECs														
**13**	21.8	329			301		137	1	1	*New compound (isomer of **F8**)*		1.00		
**14**	23.0	329			301	193	137	1	1	*New compound (isomer of **F8**)*		0.50		
**15**	23.3	329			301		137	1	1	*New compound (isomer of **F8**)*		0.28		
**16**	24.2	329			301		137	1	1	*5,6:7,8-Diedroxy-2-[2-(3-hydroxy-4-methoxy)phenylethyl]-5,6,7,8-tetrahydrochromone*	**F8**	0.47		
**17**	24.6	329			301		137	1	1	*New compound (isomer of **F8**)*		0.02		
**18**	42.0	313			285		121	1		*Oxidoagarochromones B*	**F9**	4.85		
**19**	42.9	283			255					*Oxidoagarochromones A*	**F10**	0.89		0.21

CM: chromone moiety; BM: benzyl moiety; RFC: Reference compound; RC: Relative content.

**Table 3:** The MS characterization and identified or characterized results of FTPECs from S1 to S3 by HPLC/DAD/ESI/MS^2^.

No.	t_R_ (min)	[M + H]^+^ (*m/z*)	Fragment Ions (*m/z*)		CM		BM		Identification	RFC	RC (%)		
			CM	BM	OCH_3_	OH	OCH_3_	OH			S1	S2	S3
**20**	26.1	329		137		2	1	3′-	*6,8-Dihydroxy-2-[2-(3-hydroxy-4-methoxyl)phenylethyl]-chromone*		0.28		
**21**	34.2	283	177	107		6-		4′-	*6-Hydroxy-2-[2-(4-hydroxy)phenylethyl]chromone*	**F11**		8.6	0.03
**22**	35.0	313	177	137		1	1	4′or 2′-	*6-Hydroxy-2-[2-(3-methoxyl-4-hydroxy)phenylethyl]-chromone*			0.26	0.26
**23**	36.8	313		137		6-	4′-	3′-	*6-Hydroxy-2-[2-(3-hydroxy-4-methoxyl)phenylethyl]-chromone*	**F12**	0.66	0.23	0.41
**24**	38.4	313		121		6,8-	4′-		*6,8-Dihydroxy-2-[2-(4-methoxy)phenylethyl]chromone*	**F13**	0.66	0.87	2.32
**25**	39.5	283	192	91		6,8-			*6,8-Dihydroxy-2-(2-phenylethyl)chromone*	**F14**		0.93	1.81
**26**	41.3	283	177	107		6-		2′-	*6-Hydroxy-2-[2-(2-hydroxy)phenylethyl]chromone*	**F15**		0.05	
**27**	42.7	313		121		6,7-	4′-		*6,7-Dihydroxy-2-[2-(4-methoxy)phenylethyl]chromone*	**F16**	2.80	0.46	0.45
**28**	44.0	283	192	91		2			*New compound (isomer of **F14**)*		1.33	0.52	
**29**	59.1	343		137	6-	5-	3′-	4′-	*5-Hydroxy-6-methoxy-2-[2-(3-methoxy-4-hydroxy)phenyl-ethyl]chromone*	**F27**	1.18		
**30**	66.3	313		121		2	1		*5,8-Dihydroxy-2-[2-(4-methoxy)phenylethyl]chromone*			1.82	
**31**	68.5	283	192	91		2			*5,8-Dihydroxy-2-(2-phenylethyl)chromone*			0.78	
**32**	43.1	357	220	137	6,7-		4′-	3′-	*6,7-Dimethoxy-2-[2-(3-hydroxy-4-methoxyl)phenylethyl]-chromone*	**F17**		0.46	
**33**	46.8	267	161	107				1	*2-[2-(4-Hydroxy)phenylethyl]chromone*		0.27	0.21	0.20
**34**	47.5	297	161	137			1	4′-	*2-[2-(3-Methoxy-4-hydroxy)phenylethyl]chromone*	**F18**	0.22		
**35**	48.5	297	191	107	1			2′ or 4′-	*6-Methoxy-2-[2-(4-hydroxy)phenylethyl]chromone or new compound*			0.16	
**36**	49.2	267	176	91		8-			*8-Hydroxy-2-(2-phenylethyl)chromone*	**F19**		0.78	1.16
**37**	50.3	297		121		1	1		*6-Hydroxy-2-[2-(4-methoxy)phenylethyl]chromone or new compound*		0.16	0.04	1.16
**38**	51.4	327		121	6-	7-	4′-		*6-Methoxy-7-hydroxy-2-[2-(4-methoxy)phenylethyl]-chromone*	**F20**	1.27	1.57	2.55
**39**	52.3	327	191	137	6-		3′-	4′-	*6-Methoxy-2-[2-(3-methoxy-4-hydroxy)phenylethyl]-chromone*	**F22**	2.88	0.85	1.88
**40**	53.0	297	206		1	1			*6-Hydroxy-7-methoxy-2-(2-phenylethyl)chromone*			1.74	2.32
**41**	54.3	327		137	6-		4′-	3′-	*6-Methoxy-2-[2-(3-hydroxy-4-methoxy)phenylethyl]-chromone*	**F23**	5.43	2.06	1.98
**42**	54.9	267	161	107				2′-	*2-[2-(2-Hydroxy)phenylethyl]chromone*	**F24**		1.06	
**43**	55.8	297		121		1	1		*6-Hydroxy-2-[2-(4-methoxy)phenylethyl]chromone or new compound*	**F25**	4.25	5.36	8.17
**44**	57.6	267	176	91		6-			*6-Hydroxy-2-(2-phenylethyl)chromone*	**F26**	1.42	6.64	8.17
**45**	58.7	297	191	107	1			2′ or 4′-	*6-Methoxy-2-[2-(4-hydroxy)phenylethyl]chromone or new compound*			0.27	
**46**	78.2	357		121	2	1	1		*New compound (two methoxyls and one hydroxyl on CM, one methoxyl on BM)*			1.00	
**47**	78.7	327		121	6-	5-	4′-		*5-Hydroxy-6-methoxy-2-[2-(4-methoxy)phenylethyl]-chromone*	**F31**	4.31	2.79	3.47
**48**	80.3	297	206	91	1	1			*5-Hydroxy-6-methoxy-2-(2-phenylethyl)chromone*		1.36	2.58	2.23
**49**	62.5	341		121	2		1		*6,7-Dimethoxy-2-[2-(4-methoxy)phenylethyl]chromone*		5.67	5.06	4.43
**50**	63.3	341		151	1		2		*New compound (one methoxyl on CM, two methoxyls on BM)*		1.28	0.51	
**51**	64.8	311	220	91	6,7-				*6,7-Dimethoxy-2-(2-phenylethyl)chromone*	**F28**	5.58	6.41	11.93
**52**	71.7	281		121			4′-		*2-[2-(4-Methoxy)phenylethyl]chromone*	**F29**	0.62	0.98	1.18
**53**	73.2	281	190	91	1				*6-Methoxy-2-(2-phenylethyl)chromone or new compound*		0.17		
**54**	73.4	251	160	91					*2-(2-phenylethyl)chromone*	**F30**	0.17	1.05	1.52
**55**	74.7	311		121	1		1		*6-Methoxy-2-[2-(3-methoxy)phenylethyl]chromone or 6-methoxy-2-[2-(4-methoxy)phenylethyl]chromone*			4.89	6.59
**56**	76.3	281	190	91	1				*6-Methoxy-2-(2-phenylethyl)chromone or new compound*		2.28	10.6	9.34

CM: chromone moiety; BM: benzyl moiety; RFC: Reference compound; RC: Relative content.2.3.1. Structural Analysis of THPECs

**Table 4:** The statistical results of 2-(2-phenylethyl)chromone derivatives.

N(RC/%)	DEPECs	EPECs	Total (E)	THPECs	FTPECs	Total (all)
S1	7 (8.01%)	4 (12.58%)	11 (20.59%)	3 (1.58%)	23 (44.25%)	37 (66.42%)
S2	0 (0%)	5 (8.07%)	5 (8.07%)	5 (1.73%)	33 (71.59%)	43 (81.39%)
S3	1 (0.21%)	2 (2.96%)	3 (3.17%)	3 (2.47%)	23 (73.56%)	29 (79.20%)

N: number; RC: Relative content; E: DEPECs and EPECs.
